# Exploring Australian pharmacists’ perceptions and practices towards reducing the risk of medicines-related harm in aged care residents

**DOI:** 10.3389/fphar.2023.1131456

**Published:** 2023-03-01

**Authors:** Sheraz Ali, Colin M. Curtain, Gregory M. Peterson, Mohammed S. Salahudeen

**Affiliations:** School of Pharmacy and Pharmacology, College of Health and Medicine, University of Tasmania, Hobart, TAS, Australia

**Keywords:** medicines-related harm, pharmacist, perceptions, practices, aged care, older resident, Australia, embedded pharmacist

## Abstract

**Background:** Older people living in residential aged care facilities frequently experience medicines-related harm. Evidence regarding the perception and practices towards reducing these harms may facilitate the development of customised educational programs for pharmacists providing services in RACFs.

**Objective:** To explore Australian pharmacists’ opinions and practices towards reducing the risk of medicines-related harm in aged care residents.

**Methods:** An online survey was developed based on a literature review, expert opinion, and feedback from pharmacists providing services in RACFs. A web link for the survey was shared *via* professional pharmacy organisations and social media groups with Australian pharmacists providing services in RACFs.

**Results:** A total of 209 pharmacists participated in the survey. Of these, 76% (*n* = 158) were residential medication management review embedded pharmacists, and 24% (*n* = 51) were supply pharmacists for RACFs. Most pharmacists believed that medicines-related harm is common in residents (*n* = 174, 83%), yet few agreed that pharmacists have enough time to participate in medicines-related harm reduction services (*n* = 60, 28%). There was a high level of agreement regarding the key risk factors (e.g., inappropriate medicines, anticholinergic drug use, and transitions of care) and potential strategies (e.g., embedded pharmacists in RACFs, educating aged care staff, and collaborative pharmacist-led medication reviews) for reducing medicines-related harm in residents.

**Conclusion:** Pharmacists agreed that older residents often experience medicines-related harm, but they did not frequently participate in medicines-related harm reduction services. Initiatives to engage pharmacists in team-based harm reduction services and educate aged care staff regarding safe medication management may improve residents’ safety and health outcomes.

## 1 Introduction

Medicines play an integral role in disease management, yet can be associated with significant problems, especially in vulnerable older people (Elliott RA, 2014). Older people are more prone to the side effects of medicines due to age-related changes in pharmacokinetics and pharmacodynamics, the presence of co-morbidities, and the use of multiple medicines (Eshetie et al., 2018). Medicines-related harm is any injury resulting from the use of medicines (Gyllensten et al., 2013; EUROmediCAT, 2015); it is recognised as a major healthcare concern in older people and places a substantial economic burden on the health system (Cahir et al., 2019). Medicines-related harm is a common cause of hospitalisations in older people, and the annual cost of medication-related hospital admissions is about $1.4 billion in Australia (Parameswaran Nair et al., 2016; Lim et al., 2022). Potentially inappropriate medication (PIMs) are defined as “medications that should be avoided due to their risk which outweighs their benefit and when there are equally or more effective but lower risk alternatives available” (Alhawassi et al., 2019). In particular, the risk of medicines-related harm is high in residential aged care facilities (RACFs) because of, among other reasons, the use of PIMs and the overall magnitude of medicines usage (Ali et al., 2021). A review indicated that half of older residents in RACFs are prescribed at least one PIM (Morin et al., 2016).

Pharmacists are recognised as experts in pharmacotherapy and could potentially prevent medicines-related harm in older people (Lee et al., 2015). A pharmacist-conducted medication review is a common intervention for reducing medicines-related harm in aged care residents (Ali et al., 2021). A previous study in Australia reported that the provision of pharmacist-led medication reviews appears to decrease the risk of mortality in residents (Sluggett et al., 2022). Evidence also indicates that the lack of accessibility to pharmacists is an important factor affecting the rational use of medicines in RACFs (Al-Jumaili and Doucette, 2017). However, the services offered by pharmacists to RACF patients and the impact of these services are still not clear (Lee et al., 2019). Medication management in RACFs is an ongoing concern (Kosari et al., 2021) and in Australia the final report of the Royal Commission into Aged Care Quality and Safety highlighted significant problems with the treatment of older people in RACFs, particularly the overuse of antipsychotic drugs (Royal Commission into Aged Care Quality and Safety, 2021). The report made numerous recommendations about the increased participation of pharmacists in medication management in aged care (Royal Commission into Aged Care Quality and Safety, 2021).

Previous studies in Australian RACFs focused on determining pharmacists’ views towards antibiotic prescribing (Lim et al., 2014) and monitoring of psychotropics (Langford et al., 2021). Overall, there is a lack of evidence regarding perceptions and practices of pharmacists providing services in RACFs towards improving medicines use. Understanding current practices and perceptions of pharmacists providing services in RACFs towards reducing the risk of medicines-related harm may provide insights to enhance safety and mitigate medicines-related morbidity and mortality. Therefore, we aimed to investigate pharmacists’ perceptions and practices towards reducing medicines-related harm among older people living in RACFs. As a secondary objective, we compared the responses based on pharmacists providing varying services to RACFs, e.g., through the provision of Residential Medication Management Reviews (RMMRs), supplying medications to RACFs, or being an embedded/in-house pharmacist, for assessing the differences in their perceptions and practices towards reducing medicines-related harm among residents. An embedded pharmacist performs medication management (e.g., medicine reviews) and quality improvement activities (e.g., revising medicine administration protocols), and educates aged care staff regarding medicines and their use. In Australia, an accredited pharmacist, usually on a visitational basis, conducts RMMRs that facilitate the quality use of medicines and help reduce the incidence of medicines-related injury in government-funded RACFs (Pharmacy Programs Administrator, 2022). This includes sharing a medication review report with each resident’s general practitioner to encourage implementation of the pharmacist’s recommendations for improving medicines management (Pharmaceutical Society of Australia, 2019). An RMMR pharmacist provides services to RACFs remotely or in a visiting capacity. In Australia, the aged care pharmacist services are implemented and organised through a funding provided by the Australian government (Kosari et al., 2021). All RACFs do not have a RMMR pharmacist as the level of pharmacy support could vary between RACFs and is subject to the discretion of the RACF management and the specific needs of the residents (Kosari et al., 2021). Similarly, some RACFs can choose to contract with a RMMR pharmacist for regular medication reviews, while others may rely on other healthcare professionals to manage residents’ medication needs. The role of Australian aged care supply pharmacists is to ensure that older people in RACFs receive appropriate and safe medication management (Pharmaceutical Society of Australia, 2019). Moreover, the supply pharmacists are responsible for managing the supply and dispensing of medicines, as well as ensuring that aged care residents receive the right medicine at the right time. This includes monitoring the effectiveness of medicines, identifying any potential medicines-related harm, and ensuring that residents receive appropriate support and advice to manage their medicines (Tait et al., 2021). A supply pharmacist also works closely with other healthcare professionals, including doctors and nurses, to ensure that patients receive comprehensive and coordinated care (Tait et al., 2021).

## 2 Methods

A cross-sectional national survey was conducted between February 2022 and August 2022. Pharmacists across Australia providing clinical or supply services to RACFs were the target population.

### 2.1 Development of the survey

An initial draft of the survey was generated based on a literature review, expert opinion, and feedback collected from pharmacists providing services to RACFs. The survey comprised questions on demographics, pharmacists’ extent of agreement with statements regarding medicines-related issues and practice considerations, and perceptions towards risk factors for medicines-related harm and potential strategies for reducing medicines-related harm in aged care residents. Question types included Likert scales and free-text boxes. The face and content validity of the draft questionnaire was determined through a pilot sample of 10 registered pharmacists. Based on feedback, the questionnaire was reviewed to ensure it was easy to understand and complete. It was designed to be completed within 15 min.

### 2.2 Sample size and recruitment of pharmacists

In 2020, the total number of RACFs in Australia was 3,300 (Australian Institute of Health and Welfare, 2020). An assumption was made that at least one pharmacist would provide services to each RACF. With a 95% confidence interval and a 5% margin of error, a sample size of 345 was estimated as being required for the survey (Raosoft Inc, 2004). A web link for the online survey using SurveyMonkey^®^ was shared with pharmacists *via* Australian pharmacy organisations (Australian Association of Consultant Pharmacy, Pharmacy Guild of Australia, Professional Pharmacists Australia, and Pharmaceutical Society of Australia), Pharmacy Daily (an online publication), and social media groups (LinkedIn, Facebook, and Twitter). An information sheet was on the cover page of the survey to provide general information to the potential study participant, including eligibility such as providing any type of services to RACFs. Completion of the survey was deemed as implied consent. We opted for the “Off” setting under multiple responses in SurveyMonkey^®^ portal, which prevents the survey from being taken multiple times from the same device. Further, we added an additional note “If you have already taken this survey, then you do not need to submit it again” on the cover page of the survey to prevent multiple responses from the same pharmacist. All participants who completed the survey were entered in a draw to receive one of two AUD$100 gift cards. All returned questionnaires were reviewed for eligibility and completion.

### 2.3 Statistical analysis

Data analysis was carried out using SPSS (IBM Corp. Released 2012. IBM SPSS Statistics for Windows, version 26.0. Armonk, NY, United States: IBM Corp.) and Microsoft Office Excel 2019. The mean ± standard deviation was used to present normally distributed continuous data. Ordinal or skewed data were presented using the median [interquartile range (IQR)], and frequency (percentage) was used to report categorical variables. The study questionnaire was comprised of four main sections: Perception (13 items), Practices (6 items), Risk Factors (11 items) and Strategies (7 items), and a 5-point Likert scale (1 = Strongly disagree to 5 = Strongly agree) was used. Inferential statistics, such as a chi-square test for categorical and Mann-Whitney *U* test for non-parametric numerical variables, were used to compare responses between the RMMR/embedded and supply pharmacists. Many supply pharmacists also provide services as RMMR pharmacist after receiving case-related training and accreditation from the Australian Association of Consultant Pharmacists. The purpose of comparing these groups was to assess the differences in their perceptions and practices towards reducing medicines-related harm among aged care residents. A *p*-value of <0.05 was used as the level of significance in all analyses.

### 2.4 Ethics

Approval was obtained from the Tasmanian Human Research Ethics Committee (Reference: H0026755). We followed the reporting guidelines of the STROBE (Strengthening the Reporting of Observational Studies in Epidemiology) statement for observational studies (von Elm et al., 2007).

## 3 Results

From February to August 2022, 209 pharmacists completed the survey. One hundred and forty-six were RMMR pharmacists and twelve were embedded pharmacists within RACFs; we combined these groups as their roles are relatively similar and distinct from supply pharmacists. The demographics of the pharmacists are detailed in Table 1. The median years of experience in providing services to RACFs was 7 years (range, 3–15 years), and 173 pharmacists (83%) were accredited to perform RMMRs. About half of the pharmacists provided services to more than three RACFs.

**TABLE 1:** Comparison of study demographics and characteristics between RMMR/embedded and supply pharmacists.

Variables	Overall	RMMR/Embedded pharmacist	Supply pharmacist	*p*-value
Number of Respondents	209	158 (75.6)	51 (24.4)	
Gender				
Male	59 (28.2)	37 (23.4)	22 (43.1)	0.007
Female	150 (71.8)	121 (76.6)	29 (56.9)	
Age, years				
20–29 years old	20 (9.6)	13 (8.2)	7 (13.7)	0.037
30–39 years old	88 (42.1)	61 (38.6)	27 (52.9)	
40–49 years old	46 (22.0)	34 (21.5)	12 (23.5)	
50–59 years old	27 (12.9)	25 (15.8)	2 (3.9)	
60 years or older	28 (13.4)	25 (15.8)	3 (5.9)	
State/territory				
New South Wales	57 (27.3)	45 (28.5)	12 (23.5)	0.020
Victoria	34 (16.3)	31 (19.6)	3 (5.9)	
Queensland	38 (18.2)	26 (16.5)	12 (23.5)	
South Australia	38 (18.2)	31 (19.6)	7 (13.7)	
Western Australia	20 (9.6)	14 (8.9)	6 (11.8)	
Tasmania	18 (8.6)	9 (5.7)	9 (17.6)	
Northern Territory	1 (0.5)	1 (0.6)	0	
Australian Capital Territory	3 (1.3)	1 (0.6)	2 (3.9)	
Geographical location of work				
Urban	114 (54.6)	88 (55.7)	26 (51)	0.807
Rural/regional	92 (44)	68 (43)	24 (47.1)	
Remote	3 (1.4)	2 (1.3)	1 (2)	
Number of RACFs served				
1	57 (27.3)	36 (22.8)	21 (41.2)	0.021
2–3	52 (24.9)	39 (24.7)	13 (25.5)	
>3	100 (47.8)	83 (52.5)	17 (33.3)	
Years of experience				
Median (IQR)	7 (3–15)	7 (3–15)	6 (3–12)	0.123*
Accredited to do RMMRs				
Yes	173 (82.8)	153 (96.8)	20 (39.2)	<0.001
No	36 (17.2)	5 (3.2)^a^	31 (60.8)	
Perform RMMRs per month				
<10	57 (27.3)	51 (32.3)	6 (11.8)	<0.001
10–20	34 (16.3)	34 (21.5)	0	
>20	55 (26.3)	55 (34.8)	0	
Not Applicable	63 (30.1)	18 (11.4)^b^	45 (88.2)	

RMMR, Residential Medication Management Reviews. All categorical data presented in *n* (%) and years of experience in median (IQR, interquartile range). Chi-square for categorical and *Mann-Whitney *U* test applied for non-parametric data. *p* < 0.05 was considered statistically significant.

^a^ They were probably accredited in the past, but they were not accredited at the time of the study.

^b^ They had conducted RMMRs, in the past, but they were not conducting RMMRs, at the time of the study.

Table 2 shows the comparison of responses between RMMR/embedded and supply pharmacists regarding perceptions, practices, risk factors and strategies in reducing medicines-related injury in RACFs. We merged the responses of the Likert scale into two categories - scoring ≤3 (disagree) and ≥4 (agree). Many of the pharmacists believed that medicines-related harms are common (*n* = 174, 83%) and only half believed that safe medication management is usually practiced in RACFs (*n* = 106, 51%). Almost all pharmacists agreed regarding the risk factors for medicines-related harm in aged care residents, such as polypharmacy, PIMs, dementia, recent drug changes, shortage of aged care staff, transitions of care, renal impairment, and specific drug use (e.g., antipsychotics and anticholinergics). A minority of respondents agreed that antibiotics (*n* = 86, 41%) and antipsychotics (*n* = 89, 42%) are usually prescribed in appropriate circumstances in older residents.

**TABLE 2:** Comparison of Percentage Responses between RMMR/embedded and supply pharmacists regarding perception, practices, risk factors & strategies in reducing medicines-related harm in aged care.

Categories	RMMR/Embedded pharmacist	Supply pharmacist	*p*-value
Statements	Agree	Agree	
Perceptions			
1. Medicines-related harms are common in aged care	134 (84.8)	40 (78.4)	0.289
2. Antipsychotics are generally prescribed in appropriate circumstances in aged care	62 (39.2)	27 (52.9)	0.085
3. Antibiotics are generally prescribed in appropriate circumstances in aged care	54 (34.2)	32 (62.7)	**<0.001**
4. Safe medication management is generally practiced in RACFs	82 (51.9)	24 (47.1)	0.548
5. RMMRs prevent medicines-related harms	142 (89.9)	40 (78.4)	**0.034**
6. During RMMRs, pharmacists’ recommendations to reduce medicines-related harms are typically accepted by general practitioners	89 (62)	24 (47.1)	0.059
7. Pharmacists identify medicines-related harms whilst supplying medications	71 (44.9)	43 (84.3)	**<0.001**
8. Enhanced collaboration between pharmacists and aged care staff reduces medicines-related harms	151 (95.6)	48 (94.1)	0.673
9. Pharmacists report possible medicines-related harms in aged care to general practitioners	130 (82.3)	39 (76.5)	0.359
10. Aged care management personnel (*e.g., administrators/managers*) are open to pharmacists’ suggestions for reducing medicines-related harms	139 (88)	33 (64.7)	**<0.001**
11. Pharmacists have enough time to participate in medicines-related harm reduction services, such as counselling and the provision of customised education in aged care	49 (31)	11 (21.6)	0.195
12. I am familiar with tools to calculate the ‘anticholinergic drug burden’	134 (84.8)	29 (56.9)	**<0.001**
13. I am familiar with the Beers criteria for potentially inappropriate medication use in older people	134 (84.8)	30 (58.8)	**<0.001**
Risk Factors			
1. Polypharmacy	153 (96.8)	50 (98)	0.654
2. Potentially inappropriate medications	151 (95.6)	49 (96.1)	0.876
3. Dementia	142 (89.9)	46 (90.2)	0.947
4. Drug changes in the preceding few months	146 (92.4)	41 (80.4)	**0.015**
5. Shortage of aged care nursing staff	149 (94.3)	42 (82.4)	**0.008**
6. Any antipsychotic use	146 (92.4)	45 (88.2)	0.356
7. Transitions of care	149 (94.3)	47 (92.2)	0.581
8. Any antibiotic use	125 (79.1)	35 (68.6)	0.124
9. Renal impairment (eGFR <30 mL/min)	144 (91.1)	44 (86.3)	0.315
10. Multi-morbidity	151 (95.6)	48 (94.1)	0.673
11. Anticholinergic drug use	149 (94.3)	49 (96.1)	0.622
Strategies			
1. Having an embedded pharmacist in the facility	130 (82.3)	46 (90.2)	0.178
2. More frequent pharmacist-led medication reviews	143 (90.5)	46 (90.2)	0.948
3. Collaborative medication reviews with a general practitioner	151 (95.6)	47 (92.2)	0.343
4. Provision of customized education to aged care staff	142 (89.9)	48 (94.1)	0.359
5. More frequent visits to the facility by general practitioners	133 (84.2)	45 (88.2)	0.478
6. More registered nurses on duty	142 (89.9)	47 (92.2)	0.630
7. Antimicrobial stewardship	143 (90.5)	45 (88.2)	0.639
Practices			
1. I routinely participate in medicines-related harm reduction services in aged care, such as counselling and the provision of educational support to aged care staff	95 (60.1)	15 (29.4)	**<0.001**
2. I routinely report adverse drug reactions to the Therapeutic Goods Administration	35 (22.2)	11 (21.6)	0.930
3. I routinely report possible medicines-related harms to general practitioners	135 (85.4)	36 (70.6)	**0.017**
4. I routinely utilize tools (*e.g., anticholinergic drug burden scale, Beer’s criteria*) that predict the risk of an aged care resident experiencing medicines-related harm	100 (63.3)	14 (27.5)	**<0.001**
5. I routinely recommend deprescribing interventions	146 (92.4)	24 (47.1)	**<0.001**
6. I communicate with prescribers if unsure about the appropriateness of any medication	134 (84.8)	45 (88.2)	0.544

RMMR, Residential Medication Management Review. Agree = Likert score 4-5. Data presented in *n* (%). Chi-square was applied. *p* < 0.05 is considered statistically significant, highlighted in bold font.

As with risk factors, the pharmacists displayed high agreement regarding potential strategies (e.g., presence of embedded pharmacists in RACFs, frequent pharmacist-led medication reviews, collaborative medication reviews with a general practitioner, and educating aged care staff) for reducing medicines-related harm in aged care residents. Enhancing collaboration between pharmacists and aged care staff was acknowledged by almost all pharmacists as a key strategy to reduce this harm in older residents (*n* = 199, 95%). Most pharmacists also agreed that RMMRs prevent medicines-related harm (*n* = 182, 87%), although only 62% of the RMMR/embedded pharmacists believed that their recommendations during RMMRs were typically accepted by general practitioners. Most pharmacists, particularly within the RMMR/embedded pharmacist group, agreed that aged care management personnel consider pharmacists’ suggestions for reducing medicines-related harm in residents (*n* = 172, 82%), but few agreed that pharmacists have enough time to participate in medicines-related harm reduction services (*n* = 60, 28%).

Only half of the pharmacists indicated that they routinely participate in harm reduction services (*n* = 110, 53%); this was lower for supply pharmacists (29%) relative to the RMMR/embedded pharmacists (60%). Also, a significantly lower proportion of the supply pharmacists reported that they were familiar with medication appropriateness tools (e.g., Beers criteria and anticholinergic drug burden) and routinely utilised these tools. Overall, very few supply pharmacists participated in harm reduction services, reported adverse drug reactions to the Australian Therapeutic Goods Administration, and utilised tools to predict the risk of medicines-related harm. There was a large difference in the proportions of supply and RMMR/embedded pharmacists (84% and 45%, respectively) who agreed that supply pharmacists detect medicines-related harms whilst supplying medicines. Most pharmacists indicated that they routinely recommend deprescribing interventions (*n* = 170, 81%); this was lower for supply pharmacists (47%) relative to the RMMR/embedded pharmacists (92%).

## 4 Discussion

The role of pharmacists in aged care is evolving globally and may be critical in reducing the incidence of medicines-related injury in aged care residents (Ali et al., 2021; Haider et al., 2021). Knowing the perceptions and current practices of pharmacists providing services in RACFs should be useful when developing interventions towards reducing the risk of medicines-related harm in older residents.

Most pharmacists agreed that medicines-related harm is highly prevalent in residents. Aged care residents take many medicines for their multiple morbidities, and it is a responsibility of all stakeholders involved in managing older residents to ensure the safe medication management (Department of Health and Aged Care, 2022). All stakeholders should follow the guiding principles for medication management in RACFs (e.g., person-centred care, medication reconciliation, and selection of medicines) issued by the Australian Government Department of Health and Ageing.

Pharmacists believed that older residents frequently experience medicines-related harm due to factors such as polypharmacy, PIMs, anticholinergic drug use and multi-morbidity. They believed increased collaboration between pharmacists and aged care staff would reduce these harms. The 2021 Australian Aged Care Royal Commission report recommended increased communication and collaboration between aged care staff and other healthcare professionals, such as general practitioners and pharmacists providing services in RACFs (Royal Commission into Aged Care Quality and Safety, 2021). In New Zealand, the collaborative pharmacist-led medication reviews with a general practitioner were associated with a significant reduction in falls and adverse effects in older residents (Ailabouni et al., 2019). However, only 62% of the RMMR/embedded pharmacists in our study believed that their recommendations during RMMRs were typically accepted by general practitioners. The infrequent uptake of pharmacists’ recommendations can act as a barrier in reducing medicines-related harm in residents; improving uptake of pharmacists’ recommendations during RMMRs improves safe and effective use of medicines (Cross et al., 2022).

Relatively few pharmacists in our study agreed that antibiotics and antipsychotics are frequently prescribed in appropriate circumstances in Australian aged care residents. The RedUSe intervention, comprising multidisciplinary case review, provision of education to the staff, and audit and feedback, has recently been introduced in RACFs to reduce inappropriate antipsychotic prescribing (Westbury et al., 2018). Antibiotic stewardship could similarly help to reduce inappropriate prescribing of antibiotics and the occurrence of antibiotic-associated harm in residents (Ali et al., 2021).

Most RMMR/embedded pharmacists believed that supply pharmacists do not usually identify medicines-related injury whilst supplying medicines. Many supply pharmacists in our study were not familiar with tools, such as the Beers criteria and anticholinergic drug burden scale, to predict the risk of medicines-related harm in aged care residents. In addition, very few supply pharmacists regularly utilised these tools or routinely engaged in harm reduction services. Similarly, a Malaysian study reported that only 27% of community pharmacists were familiar with Beers criteria and 17% frequently used this tool in practice (Foong et al., 2020). Educating supply pharmacists regarding these medication appropriateness tools may facilitate improving medication safety whilst supplying medicines. In essence, their role should not be simply restricted to providing medicines without any clinical involvement. Lack of time is the greatest barrier to pharmacist participation in medication safety services (Li et al., 2018).

In Australia, pharmacists usually provide services in RACFs *via* supplying medicines and consultancy, and these roles are executed remotely or in a visiting capacity (Sluggett et al., 2017). There is a need to physically integrate pharmacists within RACF teams (Kosari et al., 2021). Embedded pharmacists can facilitate the quality use of medicines in RACFs (McDerby et al., 2020a). In 2023, all government funded RACFs across Australia will be able to engage a part-time embedded pharmacist (Cross et al., 2022). A recent Australian study reported that pharmacist integration for 15 h each week in RACFs increased the provision of education for the aged care staff (McDerby et al., 2020b).

### 4.1 Strengths and limitations

This study is the first of its kind that explored the perceptions and practices of Australian pharmacists providing services in RACFs towards reducing the risk of medicines-related injury in residents. One of the key study limitations was that the recommended sample size was not reached, indicating that these findings may not be representative of the entire group of Australian pharmacists providing services for aged care residents.

## 5 Conclusion

Pharmacists agreed that aged care residents frequently experience medicines-related harm, but their involvement in medicines-related harm reduction services was relatively limited. Initiatives to engage pharmacists in harm reduction services and educate aged care staff regarding safe medication management may improve residents’ health outcomes. Medicines use in aged care residents can be optimised by the presence of embedded pharmacists in a team environment, provision of education to aged care personnel, and collaborative medication reviews with general practitioners. Ideally, supply pharmacists would also play a greater role in accepting responsibility for the risks posed by the medicines they provide to RACFs. Future research is needed to assess the impact of educating aged care staff about safe medication management on residents’ health outcomes.

## Data Availability

The original contributions presented in the study are included in the article/Supplementary Material, further inquiries can be directed to the corresponding author.
