# Supplementary material for: Exploring Australian pharmacists’ perceptions and practices towards reducing the risk of medicines-related harm in aged care residents
Source: Front Pharmacol. 2023 Mar 1;14:1131456. doi: 10.3389/fphar.2023.1131456 (PMC10014529; doi:10.3389/fphar.2023.1131456)
Supplement: Supplementary file 1 [file DataSheet1.PDF]

Pharmacists' perceptions and practices towards reducing the risk of medicines-related harm in aged care residents

Note:

If you have already taken this survey, then you do not need to submit it again.

Survey Questionnaire

Demographics

What is your gender

- Male
- Female
- Non-binary
- Prefer not to answer

What is your age?

- 20-29 years old
- 30-39 years old
- 40-49 years old
- 50-59 years old
- 60 years or older

Where do you reside?

- South Australia
- New South Wales
- Victoria
- Queensland
- Western Australia
- Tasmania
- Northern Territory
- Australian Capital Territory

Where is your principal employment location

- Urban
- Rural/regional
- Remote

Please indicate your **role in aged care** (You can select more than one option)

- Provision of Residential Medication Management Reviews (RMMRs)

- Supplying medications to RACF(s)
- Embedded or onsite residential care pharmacist
- Other (please specify) ______________________

How many RACFs do you service?

- 1
- 2 or 3
- >3

Please indicate your years of experience as a pharmacist **providing services to aged care** <u>(Free Text)</u>

Are you accredited to do RMMRs?

- Yes
- No

How many RMMRs do you typically perform <u>each month</u>?

- Not applicable (do not conduct RMMRs)
- < 10
- 10-20
- > 20

When considering the RACF(s) that you service,

Please indicate your extent of agreement with each of these statements regarding medicines-related issues and practice considerations

**Statement**	**Strongly agree**	**Agree**	**Neutral**	**Disagree**	**Strongly disagree**
Medicines-related harms are common in aged care					
Antipsychotics are generally prescribed in appropriate circumstances in aged care					
Antibiotics are generally prescribed in appropriate circumstances in aged care					
Safe medication management is generally practised in RACFs					
RMMRs prevent medicines-related harms					
During RMMRs, pharmacists' recommendations to reduce medicines-related harms are					

typically accepted by general practitioners					
Pharmacists’ identify medicines-related harms whilst supplying medications					
Enhanced collaboration between pharmacists and aged care staff reduces medicines-related harms					
Pharmacists report possible medicines-related harms in aged care to general practitioners					
Aged care management personnel (*e.g. administrators/managers*) are open to pharmacists’ suggestions for reducing medicines-related harms					
Pharmacists have enough time to participate in medicines-related harm reduction services, such as counselling and the provision of customised education in aged care					
I routinely participate in medicines-related harm reduction services in aged care, such as counselling and the provision of educational support to aged care staff					
I routinely report adverse drug reactions to the Therapeutic Goods Administration					
I routinely report possible medicines-related harms to general practitioners					
I am familiar with tools to calculate the ‘anticholinergic drug burden’					
I am familiar with the Beers criteria for potentially inappropriate medication use in older people					
I routinely utilise tools (*e.g. anticholinergic drug burden scale, Beers criteria*) that predict the risk of an aged care resident experiencing medicines-related harm					
I routinely recommend deprescribing interventions					
I communicate with prescribers if unsure about the appropriateness of any medication					

Please rate the importance of each of the following 'risk factors' for medicines-related harms in aged care residents.

Risk factor	Very important	Important	Neutral	Unimportant	Very unimportant
Polypharmacy					
Potentially inappropriate medications					
Dementia					
Drug changes in the preceding few months					
Shortage of aged care nursing staff					
Any antipsychotic use					
Transitions of care					
Any antibiotic use					
Renal impairment (eGFR <30 mL/min)					
Multi-morbidity					
Anticholinergic drug use					

Please rate the importance of each of the following potential strategies for reducing medicines-related harms in aged care residents?

Potential strategy	Very important	Important	Neutral	Unimportant	Very unimportant

Having an embedded pharmacist in the facility					
More frequent pharmacist-led medication reviews					
Collaborative medication reviews with a general practitioner					
Provision of customised education to aged care staff					
More frequent visits to the facility by general practitioners					
More registered nurses on duty					
Antimicrobial stewardship					

Please feel free to suggest any other strategies for reducing medicines-related harms in aged care residents____
